# NURSING HOME QUALITY RATING ASSOCIATIONS WITH PAIN IN NURSING HOME RESIDENTS WITH DEMENTIA

**DOI:** 10.1093/geroni/igae098.1388

**Published:** 2024-12-31

**Authors:** Sorah Levy, Barbara Resnick, Sarah Holmes

**Affiliations:** University of Maryland School of Nursing, Baltimore, Maryland, United States; University of Maryland School of Nursing, Baltimore, Maryland, United States; University of Maryland School of Nursing, Baltimore, Maryland, United States

## Abstract

Pain is highly prevalent among nursing home (NH) residents with dementia. Estimates suggest 35-80% of NH residents with dementia experience pain. Pain had been identified as a quality indicator used to measure NH performance. The Center for Medicare and Medicaid Services (CMS) Five-Star Quality Rating System is intended to inform the public of NH quality metrics. There should, therefore, be evidence that facilities with higher star ratings provide better pain management. This cross-sectional secondary data analysis used baseline data from an implementation study testing the impact of an intervention to manage behavioral symptoms in NH residents with dementia. Generalized linear mixed models (GLMM) were used to test the relationship between CMS five-star rating and pain level controlling for resident demographics and clinical characteristics. The sample included 553 residents with dementia living in 55 NHs. On average, NHs in the sample had a five-star rating of 3.5 (SD= 1.3) and pain was observed in 22.6% (n=125) of the sample. Five-star rating was significantly associated with having pain (p=0.041) such that the odds of having pain for residents living in a 5-star facility decreased by 87.8% (OR= 0.122, 95% CI: 0.02- 0.92) relative to a 1-star facility. Findings from this study demonstrate that quality of care indicated by star rating is associated with better pain management. It may be particularly important to focus on pain management in NHs with low star ratings.

